# Sacubitril/Valsartan and Ivabradine Attenuate Left Ventricular Remodelling and Dysfunction in Spontaneously Hypertensive Rats: Different Interactions with the Renin–Angiotensin–Aldosterone System

**DOI:** 10.3390/biomedicines10081844

**Published:** 2022-07-31

**Authors:** Fedor Simko, Tomas Baka, Peter Stanko, Kristina Repova, Kristina Krajcirovicova, Silvia Aziriova, Oliver Domenig, Stefan Zorad, Michaela Adamcova, Ludovit Paulis

**Affiliations:** 1 Institute of Pathophysiology, Faculty of Medicine, Comenius University, 81108 Bratislava, Slovakia; dr.tomas.baka@gmail.com (T.B.); pete.stanko@gmail.com (P.S.); kristina.repova@fmed.uniba.sk (K.R.); krikratina@gmail.com (K.K.); silvia.aziriova@gmail.com (S.A.); ludovit.paulis@gmail.com (L.P.); 2 3rd Department of Internal Medicine, Faculty of Medicine, Comenius University, 83305 Bratislava, Slovakia; 3 Institute of Experimental Endocrinology, Biomedical Research Center, Slovak Academy of Sciences, 84505 Bratislava, Slovakia; stefan.zorad@savba.sk; 4 Attoquant Diagnostics, 1110 Vienna, Austria; oliver.domenig@attoquant.com; 5 Department of Physiology, Faculty of Medicine in Hradec Kralove, Charles University, 50003 Hradec Kralove, Czech Republic; adamcova@lfhk.cuni.cz; 6 Institute of Normal and Pathological Physiology, Centre of Experimental Medicine, Slovak Academy of Sciences, 81371 Bratislava, Slovakia

**Keywords:** SHR, sacubitril/valsartan, ARNI, ivabradine, remodelling, cardiac dysfunction, fibrosis, renin–angiotensin–aldosterone system, angiotensin II, angiotensin 1-7

## Abstract

This study investigated whether sacubitril/valsartan and ivabradine are able to prevent left ventricular (LV) fibrotic remodelling and dysfunction in a rat experimental model of spontaneous hypertension (spontaneously hypertensive rats, SHRs) and whether this potential protection is associated with RAAS alterations. Five groups of three-month-old male Wistar rats and SHRs were treated for six weeks as follows: untreated Wistar controls, Wistar plus sacubitril/valsartan, SHR, SHR plus sacubitril/valsartan, and SHR plus ivabradine. The SHRs developed a systolic blood pressure (SBP) increase, LV hypertrophy and fibrosis, and LV systolic and diastolic dysfunction. However, no changes in serum RAAS were observed in SHRs compared with the controls. Elevated SBP in SHRs was decreased by sacubitril/valsartan but not by ivabradine, and only sacubitril/valsartan attenuated LV hypertrophy. Both sacubitril/valsartan and ivabradine reduced LV collagen content and attenuated LV systolic and diastolic dysfunction. Sacubitril/valsartan increased the serum levels of angiotensin (Ang) II, Ang III, Ang IV, Ang 1-5, Ang 1-7, and aldosterone, while ivabradine did not affect the RAAS. We conclude that the SHR is a normal-to-low serum RAAS model of experimental hypertension. While the protection of the hypertensive heart in SHRs by sacubitril/valsartan may be related to an Ang II blockade and the protective Ang 1-7, the benefits of ivabradine were not associated with RAAS modulation.

## 1. Introduction

Left ventricular (LV) hypertrophy in hypertension is considered to be a compensatory reaction to a chronically increased haemodynamic burden. LV mass enlargement supports the heart’s performance without increasing wall tension. However, a hypertensive heart is associated with fibrotic rebuilding of the LV, resulting in a deterioration of cardiac function and a worsening prognosis. It is generally believed that curbing pathological cardiac remodelling reduces the transition from a hypertensive heart to heart failure (HF). Thus, the search for novel therapeutic strategies against the consequences of haemodynamic overload-induced cardiac remodelling in various models of experimental hypertension and in clinical conditions is unremitting [1]. Hypertensive heart disease involves the structural remodelling of the musculature and collagenous and non-collagenous matrix. Myocardial hypertrophy is determined by pressure or volume overload, which induces the compensatory growth of cardiomyocytes. The structural homogeneity may be disturbed by the imbalance of two groups of substances: by increased levels of angiotensin II, aldosterone, endothelin, and catecholamines which represent stimulators of pathologic growth with fibrocyte proliferation and an overabundance of collagen; or by a reduced production of nitric oxide (NO), natriuretic peptides, bradykinin, and prostaglandins with the opposite effect on growth and proliferation. The absolute or relative overproduction of angiotensin (Ang) II and aldosterone governs the development of pathologic fibrosis associated with deteriorated heart function and rhythm disturbances [2,3]. Thus, blocking the renin–angiotensin–aldosterone system (RAAS) by angiotensin-converting enzyme (ACE) inhibitors, angiotensin II type 1 receptor (AT1R) blockers, or aldosterone receptor blockers enables the attenuation of the vasoconstrictor, pro-inflammatory, and pro-proliferative actions.

During the past decade, two novel approaches to HF management with different mechanisms of action have been introduced. Neprilysin is an enzyme expressed in the cell membrane of various tissues that splits atrial and brain natriuretic peptides (ANP and BNP, respectively). The inhibition of neprilysin by sacubitril enhances circulating ANP and BNP levels with vasodilative, diuretic, and antiproliferative actions. Since neprilysin’s substrates include both natriuretic peptides (NP) and Ang II, its inhibition increases not only the level of beneficial NP but also the concentration of adverse Ang II, potentially counterbalancing the desirable vasodilative effects of NP. To avoid this, sacubitril, an inhibitor of neprilysin, was combined with the AT1R blocker valsartan to attenuate Ang II effects [4,5]. The PARADIGM-HF study involving heart failure patients with systolic dysfunction showed that the combination of neprilysin inhibition by sacubitril and the AT1R blocker valsartan, i.e., sacubitril/valsartan (ARNI), reduced morbidity and mortality more effectively than the ACE inhibitor enalapril [6]. Thus, ARNI is becoming the cornerstone of HF therapy. Moreover, in the PARALLAX trial comprising HF patients with a preserved LV ejection fraction, sacubitril/valsartan resulted in a significantly greater decrease in plasma N-terminal pro-brain natriuretic peptide levels compared with a standard treatment affecting the renin–angiotensin system [7]. Thus, the combination of neprilysin with a renin–angiotensin system blockade may be of potential benefit in hearts with not only systolic but also diastolic LV dysfunction.

Ivabradine is a selective inhibitor of the I_f_ current in the sinoatrial node, which is responsible for pacemaking. Ivabradine reduces the heart rate (HR) without the negative inotropic effect inherent to beta-blockers. In the SHIFT study, ivabradine decreased the composite end-point of mortality and hospitalisations for HF, and it is recommended for patients with systolic HF and a HR above 70 bpm despite treatment with or in case of intolerance of beta-blockers [8].

It is generally accepted that cardiovascular protection is achieved by interfering with the excessive neurohumoral activation seen in chronic HF. Indeed, modulation of the RAAS, whose chronic activation induces a pathologic remodelling of the target organs, is pivotal in HF management. Moreover, neprilysin activity is linked to RAAS modulation: while neprilysin participates in Ang I degradation, ANP and BNP inhibit the release of renin [9].

However, data regarding the complex interference of ARNI or ivabradine with the RAAS are sparse. Thus, the aim of this study was to show in a rat experimental model of spontaneous hypertension (spontaneously hypertensive rats, SHRs) whether ARNI or ivabradine are able to protect a hypertensive heart and whether this potential protection is due to their interaction with the deleterious classical ACE/Ang II/AT1R pathway and the protective alternative ACE2/Ang 1-7/Mas receptor (MasR) pathway of the renin–angiotensin system.

## 2. Materials and Methods

### 2.1. Animals and Treatment

Twelve-week-old male Wistar rats and age- and weight-matched male SHRs (Department of Toxicology and Laboratory Animals Breeding, Slovak Academy of Sciences, Dobra Voda, Slovak Republic) were randomly divided into five groups (15 per group) and treated for six weeks as follows: Wistar rats with no treatment (C); Wistar rats treated with ARNI (68 mg/kg/day; Novartis, Basel, Switzerland) (ARNI); SHRs with no treatment (SHR); SHRs treated with ARNI (68 mg/kg/day) (SHR + ARNI); and SHRs treated with ivabradine (10 mg/kg/day; Servier, Suresnes, France) (SHR + IVA). The therapeutics were dissolved in drinking water and their concentration was adjusted to daily water consumption. The natural water consumption was 12–13 mL per 100 g body weight. To ensure that all of the water-therapeutics solutions were drunk by a particular rat, only 10 mL per 100 g body weight of solution was offered. The solutions were prepared by dissolving the appropriate amount of therapeutics in water, while no additional substance was added. The rats were housed in individual cages, fed a regular pellet diet ad libitum and maintained under standard laboratory conditions (12:12-h light–dark cycle, 22 ± 2 °C temperature, and 55 ± 10% humidity). The study was conducted in conformity with the Guide for the Care and Use of Laboratory Animals published by the US National Institutes of Health (NIH publication no. 85-23, revised 1996). The protocol was approved by the Ethics Committee of the Institute of Pathophysiology, Faculty of Medicine, Comenius University, Bratislava, Slovak Republic (approval number: 809/19-221/3; approval date: 23 April 2019).

Systolic blood pressure (SBP) and HR were measured twice before treatment and once a week during treatment by non-invasive tail-cuff plethysmography (Hugo-Sachs Elektronik, Freiburg, Germany). After six weeks of treatment, the rats were euthanised by isoflurane inhalation. Body weight (BW), heart weight, and left ventricular weight (LVW) were measured, and the LVW/BW ratio was subsequently calculated. LV samples were frozen at −80 °C and, later, hydroxyproline concentrations were measured. Blood samples were collected from the abdominal aorta during euthanasia. Serum obtained by centrifuging the blood samples at 2000× *g* for 15 min was stored at −80 °C for subsequent angiotensin and aldosterone analysis. 

### 2.2. Determination of Hydroxyproline in the Left Ventricle

Collagenous proteins in the LV were isolated by treating LV samples stepwise with different buffers, as described previously [10]. Briefly, CH3COOH-pepsin buffer (pH 1.4, 24 h at 4 °C) was used to extract soluble collagenous proteins, and 1.1 mol/L NaOH (45 min at 105 °C) was used to extract the remaining insoluble collagenous proteins. The hydrolysed samples were oxidised by chloramine T added to an acetate–citrate buffer at pH 6.0. After incubation for 20 min at room temperature, the reaction was stopped by adding 20 volumes of Ehrlich’s reagent to the mixture. The samples were then incubated at 65 °C for 15 min, and the hydroxyproline concentration (a marker of fibrosis) in the LV was measured in both collagenous fractions using spectrophotometry at 550 nm. The hydroxyproline content in the LV was subsequently calculated and expressed as mg per total weight of the LV.

### 2.3. Determination of Serum Angiotensins and Aldosterone Concentration and the Markers of Renin and ACE Activities

Serum samples from six animals per group that were not subject to prior echocardiography were used for angiotensin and aldosterone analyses. Equilibrium Ang peptide and aldosterone levels were determined by mass spectrometry, as described previously [11]. Briefly, the equilibrium peptide levels were stabilised by equilibration of the conditioned serum at 37 °C for 60 min. Thereafter, the stabilised samples were spiked with internal standards for each angiotensin metabolite (isotopes labelled Ang I, Ang II, Ang 1-7, Ang 1-5, Ang 2-8, and Ang 3-8) at concentrations of 200 pg/mL, and for aldosterone (deuterated aldosterone) at a concentration of 500 pg/mL. After a C18-based solid-phase extraction, the samples were analysed by LC–MS/MS using a reversed-phase analytical column (Acquity UPLC^®^ C18, Waters Corp., Milford, MA, USA) operating in line with a XEVO TQ-S triple quadrupole mass spectrometer (Waters Corp.) in MRM mode. The peptide recovery of the sample preparation (for each Ang metabolite in each sample) was corrected using internal standards. The corresponding response factors determined with appropriate calibration curves in the original sample matrix, which integrated signals exceeding a signal-to-noise ratio of 10, were used to assess Ang peptide concentrations. The Ang 1-5/Ang 1-7 ratio, a marker of Ang 1-7 cleavage to Ang 1-5, was subsequently calculated.

The marker of renin activity (RA-S) was subsequently calculated as the sum of Ang I and Ang II. Indeed, in previous studies, the sum of Ang I and Ang II obtained from the above equilibrium analysis was shown to be closely correlated with the measured renin activity, independent of species or treatment [12].

The marker of ACE activity (ACE-S) was subsequently calculated as the Ang II/Ang I ratio. It provides information about the expected ACE activity [13].

The aldosterone/Ang II ratio (AA2 ratio) was calculated to assess adrenal responsiveness following Ang II signalling resulting in the release of aldosterone [14].

### 2.4. Echocardiography

After six weeks of treatment, transthoracic echocardiography was performed on seven animals per group using a 14-MHz matrix probe (M12L) coupled with a GE Medical Vivid 7 Dimension System (GE Medical Systems CZ Ltd., Prague, Czech Republic), as described previously [15]. Briefly, the animals were anesthetised throughout the protocol by applying isoflurane (2.5% inspiratory concentration at a flow rate of 2 L/min) during spontaneous breathing. After placing the rat in the supine position on a warming pad (38 °C), the thoracic wall was shaved. The HR and body temperature were monitored throughout the protocol. To assess the LV systolic function, the LV end-systolic and end-diastolic internal diameters were measured from the anatomical M-mode images in a long-axis view using the leading-edge method. Subsequently, the left ventricular fractional shortening (LVFS) and ejection fraction (LVEF, using the Teichholz formula) were determined. To assess the LV diastolic function, the diastolic transmitral peak early (E) and late (A) filling velocities were measured from the two-dimensionally guided Doppler spectra of mitral inflow in the apical four-chamber view, and the E/A ratio was then calculated. The maximal velocities of the early (Em) and late (Am) diastolic wall movement waves at the level of the septal mitral annulus were determined by tissue Doppler imaging from the apical four-chamber view; the E/Em ratio was subsequently calculated. Echocardiography was performed by an experienced echocardiographer blinded to the group identity. All measurements were averaged over three consecutive cardiac cycles.

### 2.5. Statistical Analysis

The results are presented as means ± SEM. Data distribution was assessed by a Shapiro–Wilk normality test. A two-way, repeated-measures analysis of variance (ANOVA) followed by multiple comparisons with a Bonferroni post-hoc test was used for the statistical analysis of SBP and HR data. A one-way, two-tailed ANOVA followed by multiple comparisons with a Bonferroni post-hoc test was used for the statistical analysis of the remaining data, including the heart weights, LV hydroxyproline concentrations and contents, serum Ang and aldosterone levels, and echocardiography. The differences were considered significant if *p* < 0.05. The statistical analysis was conducted using GraphPad Prism 9 for Windows (GraphPad Software, La Jolla, CA, USA).

## 3. Results

### 3.1. Haemodynamics and Heart Weights

**Figure 1:**
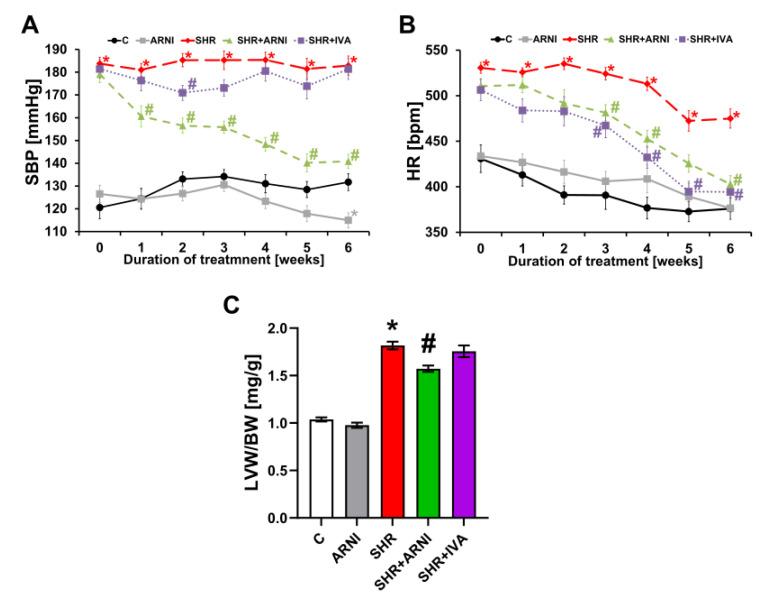
Effect of ARNI and ivabradine on systolic blood pressure (SBP) (**A**) and heart rate (HR) (**B**) throughout the experiment, and the relative weight of the left ventricle (left ventricular weight/body weight; LVW/BW) (**C**) in SHRs after six weeks of treatment. ARNI, sacubitril/valsartan; C, Wistar controls; IVA, ivabradine; SHRs, spontaneously hypertensive rats. Results are presented as means ± SEM. *n* = 15 per group. Repeated measures ANOVA (**A**,**B**) or one-way, two-tailed ANOVA (**C**) followed by multiple comparisons with a Bonferroni post-hoc test; * *p* < 0.05 vs. C; # *p* < 0.05 vs. SHR.

The SBP was 131.71 ± 3.71 mmHg in the control group, and ARNI decreased (*p* < 0.05) it by 13% after six weeks of treatment. In the SHR group, SBP was higher than in controls by 39% (182.89 ± 4.22 mmHg, *p* < 0.05 vs. C), and ARNI decreased (*p* < 0.05) it by 23%. Ivabradine did not affect SBP in SHRs (Figure 1A).

The HR was 375.93 ± 11.61 bpm in the control group, and ARNI did not affect it after six weeks of treatment. In the SHR group, the HR was higher than in controls by 26% (474.95 ± 10.53 bpm, *p* < 0.05 vs. C), and ARNI and ivabradine decreased it (*p* < 0.05) by 15% and 17%, respectively (Figure 1B).

The LVW/BW ratio was 1.04 ± 0.02 mg/g in the control group, and ARNI did not affect it after six weeks of treatment. In the SHR group, the LVW/BW ratio was higher than in controls by 75% (1.82 ± 0.04 mg/g, *p* < 0.05 vs. C), and ARNI decreased it (*p* < 0.05) by 13%. Ivabradine did not affect the LVW/BW ratio in SHRs (Figure 1C).

### 3.2. Hydroxyproline Concentration and Content in Soluble and Insoluble Collagen and Total Hydroxyproline in the Left Ventricle

**Figure 2:**
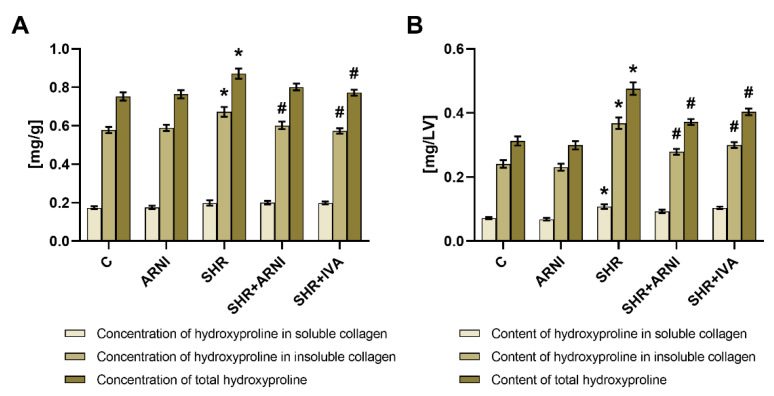
Effect of ARNI and ivabradine on hydroxyproline concentration in soluble and insoluble collagenous proteins and on the total hydroxyproline concentration (**A**), and on hydroxyproline content in the soluble and insoluble collagenous proteins, and on the total hydroxyproline content (**B**) in the left ventricle in SHRs after six weeks of treatment. ARNI, sacubitril/valsartan; C, Wistar controls; IVA, ivabradine; SHRs, spontaneously hypertensive rats. Results are presented as means ± SEM. *n* = 15 per group. One-way, two-tailed ANOVA followed by multiple comparisons with a Bonferroni post-hoc test; * *p* < 0.05 vs. C; # *p* < 0.05 vs. SHR.

The hydroxyproline concentrations in the soluble collagenous protein were 0.174 ± 0.008 mg/g and 0.199 ± 0.013 mg/g in the control and SHR groups, respectively (ns). After six weeks of treatment, none of the therapeutics affected the hydroxyproline concentration in the soluble collagenous protein (Figure 2A).

The hydroxyproline concentration in the insoluble collagenous protein was 0.578 ± 0.017 mg/g in the control group, and ARNI had no effect after six weeks of treatment. In the SHR group, the hydroxyproline concentration in the insoluble collagenous protein was higher than in controls by 16% (0.673 ± 0.025 mg/g, *p* < 0.05 vs. C), and ARNI and ivabradine decreased it (*p* < 0.05) by 11% and 15%, respectively (Figure 2A).

The total hydroxyproline concentration was 0.752 ± 0.022 mg/g in the control group, and ARNI had no effect after six weeks of treatment. In the SHR group, the total hydroxyproline concentration was higher than in controls by 16% (0.871 ± 0.026 mg/g, *p* < 0.05 vs. C), and ivabradine decreased it (*p* < 0.05) by 11%; ARNI had no significant effect (Figure 2A).

The hydroxyproline content in the soluble collagenous protein was 0.072 ± 0.003 mg/LV in the control group, and ARNI had no effect after six weeks of treatment. In the SHR group, the hydroxyproline content in the soluble collagenous protein was higher than in controls by 50% (0.108 ± 0.007 mg/LV, *p* < 0.05 vs. C), and none of the therapeutics had a significant effect (Figure 2B).

The hydroxyproline content in the insoluble collagenous protein was 0.241 ± 0.012 mg/LV in the control group, and ARNI had no effect after six weeks of treatment. In the SHR group, the hydroxyproline content in the insoluble collagenous protein was higher than in controls by 53% (0.368 ± 0.018 mg/LV, *p* < 0.05 vs. C), and ARNI and ivabradine decreased it (*p* < 0.05) by 24% and 19%, respectively (Figure 2B).

The total hydroxyproline content was 0.313 ± 0.014 mg/LV in the control group, and ARNI had no effect after six weeks of treatment. In the SHR group, the total hydroxyproline content was higher than in controls by 52% (0.476 ± 0.019 mg/LV, *p* < 0.05 vs. C), and ARNI and ivabradine decreased it (*p* < 0.05) by 22% and 15%, respectively (Figure 2B).

### 3.3. Serum Concentration of Angiotensins and Aldosterone, and the Markers of Renin and ACE Activities

**Figure 3:**
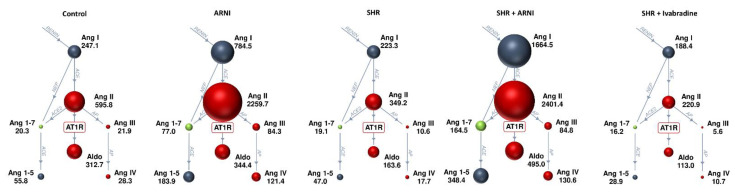
Schematic depicting the serum angiotensin and aldosterone concentrations in the study groups after six weeks of treatment. Results are presented as means. ACE, angiotensin-converting enzyme; Aldo, aldosterone; Ang, angiotensin; AP, aminopeptidase; ARNI, sacubitril/valsartan; AT1R, angiotensin II type 1 receptor; NEP, neprilysin (neutral endopeptidase); SHRs, spontaneously hypertensive rats. *n* = 6 per group.

The mean serum angiotensin and aldosterone concentrations in the study groups after six weeks of treatment are schematically depicted in Figure 3.

**Figure 4:**
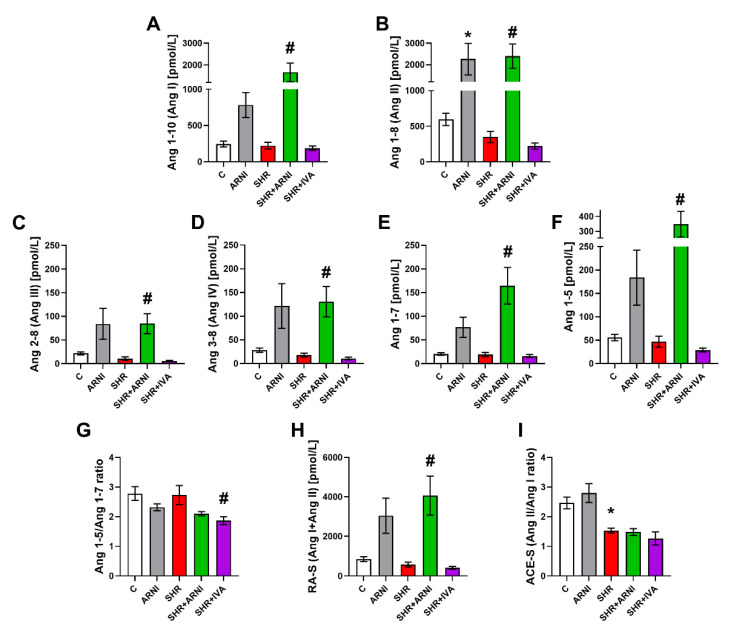
Effect of ARNI and ivabradine on the serum level of angiotensin 1-10 (Ang I) (**A**), angiotensin 1-8 (Ang II) (**B**), angiotensin 2-8 (Ang III) (**C**), angiotensin 3-8 (Ang IV) (**D**), angiotensin 1-7 (**E**), angiotensin 1-5 (**F**), angiotensin 1-5/angiotensin 1-7 ratio (**G**), marker of renin activity (RA-S; Ang I + Ang II) (**H**), and marker of angiotensin-converting enzyme activity (ACE-S; Ang II/Ang I) (**I**) in SHRs after six weeks of treatment. Ang, angiotensin; ARNI, sacubitril/valsartan; C, Wistar controls; IVA, ivabradine; SHRs, spontaneously hypertensive rats. Results are presented as means ± SEM. *n* = 6 per group. One-way, two-tailed ANOVA followed by multiple comparisons with a Bonferroni post-hoc test; * *p* < 0.05 vs. C; # *p* < 0.05 vs. SHR.

The serum equilibrium level of Ang 1-10 (Ang I) was 247.15 ± 40.13 pmol/L and 223.32 ± 47.4 pmol/L in the control and SHR groups, respectively (ns); ARNI increased it by 217% (ns) and 645% (*p* < 0.05) in the control and SHR groups, respectively (Figure 4A).

The level of Ang 1-8 (Ang II) was 595.77 ± 87.89 pmol/L and 349.22 ± 79.1 pmol/L in the control and SHR groups, respectively (ns); ARNI increased it by 279% (*p* < 0.05) and 588% (*p* < 0.05) in the control and SHR groups, respectively, after six weeks of treatment (Figure 4B).

The level of Ang 2-8 (Ang III) was 21.88 ± 3.22 pmol/L and 10.63 ± 3.88 pmol/L in the control and SHR groups, respectively (ns); ARNI increased it by 285% (ns) and 697% (*p* < 0.05) in the control and SHR groups, respectively (Figure 4C).

The level of Ang 3-8 (Ang IV) was 28.27 ± 4.49 pmol/L and 17.72 ± 4.0 pmol/L in the control and SHR groups, respectively (ns); ARNI increased it by 329% (ns) and 637% (*p* < 0.05) in the control and SHR groups, respectively (Figure 4D).

The level of Ang 1-7 was 20.33 ± 2.66 pmol/L and 19.07 ± 4.69 pmol/L in the control and SHR groups, respectively (ns); ARNI increased it by 279% (ns) and 763% (*p* < 0.05) in the control and SHR groups, respectively (Figure 4E).

The level of Ang 1-5 was 55.75 ± 6.95 pmol/L and 46.97 ± 11.54 pmol/L in the control and SHR groups, respectively (ns); ARNI increased it by 230% (ns) and 642% (*p* < 0.05) in the control and SHR groups, respectively (Figure 4F).

None of the Ang levels in SHR were significantly affected by ivabradine after six weeks of treatment (Figure 4A–F).

The Ang 1-5/Ang 1-7 ratio was 2.78 ± 0.23 and 2.73 ± 0.32 in the control and SHR groups, respectively (ns); ivabradine decreased it by 32% (*p* < 0.05) in the SHR group. ARNI had no significant effect on the Ang 1-5/Ang 1-7 ratio in either controls or SHRs (Figure 4G).

The marker of renin activity (RA-S; Ang I + Ang II) was 842.88 ± 125.38 pmol/L and 572.5 ± 125.96 pmol/L in the control and SHR groups, respectively (ns); ARNI increased it by 261% (ns) and 610% (*p* < 0.05) in the control and SHR groups, respectively. Ivabradine had no significant effect on the marker of renin activity in SHRs after six weeks of treatment (Figure 4H).

The marker of ACE activity (ACE-S; Ang II/Ang I ratio) was 2.47 ± 0.20 and 1.53 ± 0.08 in the control and SHR groups, respectively (*p* < 0.05). ARNI and ivabradine had no significant effect on ACE-S in SHRs (Figure 4I).

**Figure 5:**
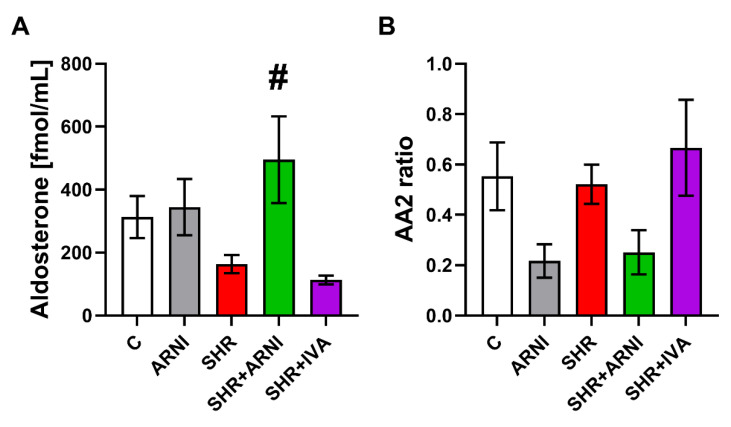
Effect of ARNI and ivabradine on the serum level of aldosterone (**A**) and the aldosterone/angiotensin II ratio (AA2 ratio) (**B**) in SHRs after six weeks of treatment. ARNI, sacubitril/valsartan; C, Wistar controls; IVA, ivabradine; SHRs, spontaneously hypertensive rats. Results are presented as means ± SEM. *n* = 6 per group. One-way, two-tailed ANOVA followed by multiple comparisons with a Bonferroni post-hoc test; # *p* < 0.05 vs. SHR.

The serum concentration of aldosterone was 312.73 ± 66.99 fmol/mL and 163.63 ± 29.02 fmol/mL in the control and SHR groups, respectively (ns); ARNI increased it by 10% (ns) and 203% (*p* < 0.05) in the control and SHR groups, respectively (Figure 5A). The aldosterone/Ang II ratio (AA2 ratio) was 0.55 ± 0.14 and 0.52 ± 0.08 in the control and SHR groups, respectively (ns); ARNI decreased it by 61% (ns) and 52% (ns) in the control and SHR groups, respectively (Figure 5B). Ivabradine had no effect on the serum concentration of aldosterone and the AA2 ratio in SHRs after six weeks of treatment (Figure 5A,B).

### 3.4. Echocardiography

**Figure 6:**
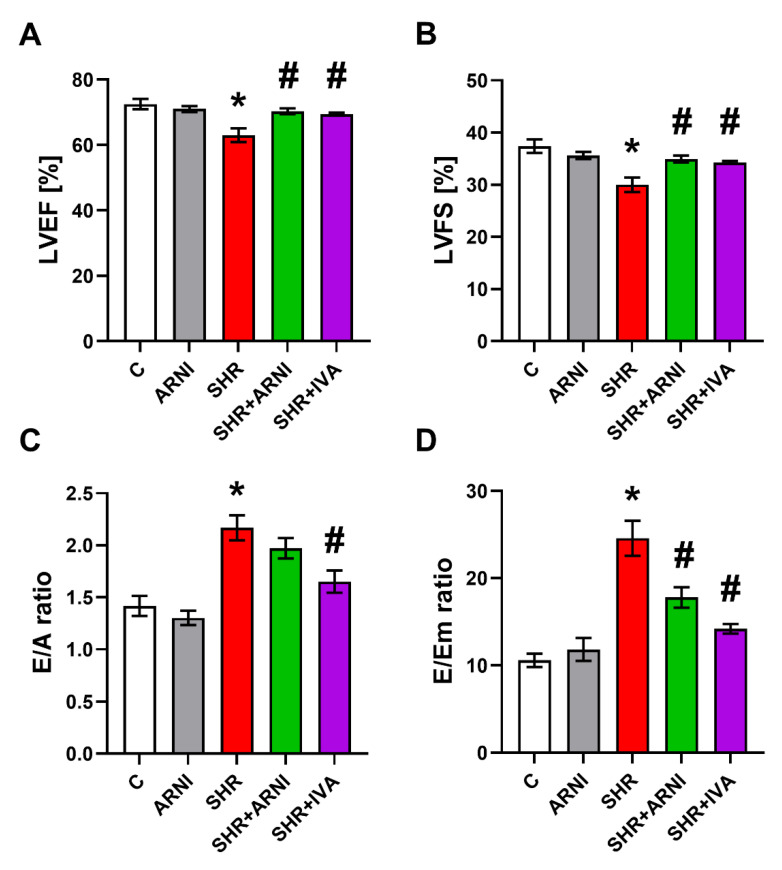
Effect of ARNI and ivabradine on left ventricular ejection fraction (LVEF) (**A**), left ventricular fractional shortening (LVFS) (**B**), the ratio of the diastolic transmitral peak early and late filling velocities (E/A ratio) (**C**), and the ratio of the diastolic transmitral peak early filling velocity and the maximal velocity of early diastolic wall movement wave at the level of mitral annulus (E/Em ratio) (**D**) in SHRs after six weeks of treatment. ARNI, sacubitril/valsartan; C, Wistar controls; IVA, ivabradine; SHRs, spontaneously hypertensive rats. Results are presented as means ± SEM. *n* = 7 per group. One-way, two-tailed ANOVA followed by multiple comparisons with a Bonferroni post-hoc test; * *p* < 0.05 vs. C; # *p* < 0.05 vs. SHR.

The LVEF was 72.52 ± 1.55% in the control group, and ARNI had no effect after six weeks of treatment. In the SHR group, the LVEF was lower than controls by 13% (63.0 ± 2.12%, *p* < 0.05 vs. C), and ARNI and ivabradine increased it (*p* < 0.05) by 12% and 10%, respectively (Figure 6A).

The LVFS was 37.38 ± 1.29% in the control group, and ARNI had no effect after six weeks of treatment. In the SHR group, the LVFS was lower than controls by 20% (30.0 ± 1.39%, *p* < 0.05 vs. C), and ARNI and ivabradine increased it (*p* < 0.05) by 17% and 14%, respectively (Figure 6B).

The E/A ratio was 1.42 ± 0.1 in the control group, and ARNI had no effect after six weeks of treatment. In the SHR group, the E/A ratio was higher than controls by 53% (2.17 ± 0.12%, *p* < 0.05 vs. C), and ivabradine decreased it (*p* < 0.05) by 24%. ARNI had no significant effect on the E/A ratio in SHRs after six weeks of treatment (Figure 6C).

The E/Em ratio was 10.58 ± 0.76 in the control group, and ARNI had no effect after six weeks of treatment. In the SHR group, the E/Em ratio was higher than controls by 132% (24.58 ± 2.01, *p* < 0.05 vs. C), and ARNI and ivabradine decreased it (*p* < 0.05) by 28% and 42%, respectively (Figure 6D).

## 4. Discussion

The effects of the neprilysin inhibitor/AT1R blocker sacubitril/valsartan (ARNI) and ivabradine on SBP, HR, myocardial remodelling, LV systolic and diastolic function, and the RAAS were investigated in SHRs. 

The SHR, a commonly employed rat experimental model of spontaneous hypertension, mimics primary hypertension with target organ damage in humans. The mechanisms underlying the development of primary hypertension are complex and comprise several potential players. Endothelial dysfunction in conduit and resistance arteries is frequently considered to contribute to the BP increase in the SHR. However, disturbed endothelial function has been described mainly in aged but not young SHRs, suggesting that endothelial dysfunction is more a consequence than a cause of elevated BP [16]. Data regarding the participation of the RAAS in hypertension pathophysiology in the SHR are driven mainly by the impact of targeted inhibition of the presumably deleterious classical ACE/Ang II/AT1R pathway [17] or by activation of the supposedly beneficial alternative ACE2/Ang 1-7/MasR pathway [18,19]. Data characterising the RAAS in the untreated SHR varies considerably in different laboratories. The serum level of renin, Ang II [20], and aldosterone [20,21], AT1R expression in mesenteric and coronary arteries [19], and heart expression of Mas-related G protein-coupled receptor D (MrgD) were higher in SHRs compared to Wistar rats, while the MasR expression in arteries was lower in SHRs [19]. Moreover, SHRs showed an altered circadian gene expression affecting the transcriptional regulation of clock-controlled genes for aldosterone and corticosterone [22]. In our experiment with three-month-old male SHRs, the levels of Ang I (Ang 1-10), Ang II (Ang 1-8), Ang III (Ang 2-8), Ang IV (Ang 3-8), Ang 1-7, and Ang 1-5 did not show significant changes compared to Wistar controls, corresponding with the unchanged marker of renin activity (RA-S). The trend towards reduced Ang II levels, albeit non-significant, corresponded with the decreased marker of ACE activity (ACE-S), calculated as the Ang II/Ang I ratio, and with the trend toward serum aldosterone concentration reduction. These data suggest that SHR is a normal-to-low renin and normal-to-low angiotensin/aldosterone model of hypertension. Thus, the underlying mechanism of hypertension and target organ damage in the SHR remains elusive. Importantly, increased renal sympathetic activity has been previously reported in the SHR [23]. Indeed, in our experiment, HR was significantly elevated in SHRs by approximately 100 bpm during the entire course of the six-week experiment, indicating activation of the sympathetic nervous system. In previous experiments, renal sympathetic denervation reduced intrarenal norepinephrine, the renal tissue protein of Ang II, aldosterone, and AT1R [24], and ameliorated renal fibrosis and dysfunction along with the delayed onset of hypertension in the SHR [25]. These results correspond with the findings of the higher activity of tyrosine hydroxylase, the rate-limiting enzyme in the synthesis of catecholamines, observed in the heart and kidney of SHRs compared with Wistar rats [26]. The above data suggest that sympathetic system activation plays a crucial role in BP elevation in the SHR. Along with sympathetic activation, the local renin–angiotensin system may also be activated in the kidney [25], brain [21], or other organs. In addition, other neurohumoral alterations, such as an elevated endothelin 1 level [20] or reduced endothelial nitric oxide synthase (eNOS) activity and nitric oxide (NO) bioavailability [16,25], may also contribute to BP elevation in the SHR. Furthermore, hypertensive encephalopathy due to a higher mineralocorticoid receptor expression and their activation by endogenous corticosterone may participate in hippocampal neuroinflammation, which may potentially contribute to BP dysregulation and hypertension [27].

In our study, the dual inhibitor of the endopeptidase neprilysin and the AT1R, sacubitril/valsartan (ARNI), significantly reduced systolic BP as well as LV mass in SHRs after six weeks of treatment. Moreover, ARNI significantly reduced the LV concentration of insoluble collagen, and numerically also the total collagen, and significantly decreased their LV contents. ARNI completely prevented the deterioration of LV systolic function and attenuated the deterioration of diastolic function in SHRs. This anti-remodelling nature of ARNI is in agreement with data from other laboratories and large clinical studies. Sacubitril/valsartan prevented myocardial fibrosis and remodelling and improved cardiac function after myocardial infarction in mice [28] and rats [29,30], and in streptozotocin-induced diabetic hearts in mice [31]; it reduced cardiomyocyte size in Ang II-induced cardiac hypertrophy in mice [32], attenuated LV fibrosis and dysfunction in high-salt diet-induced diastolic dysfunction in rats [33], and reduced BP and prevented stroke in stroke-prone hypertensive rats [34]. A meta-analysis of clinical studies from 2010 to 2019 revealed that ARNI exerted reverse remodelling in terms of reduced LV size and hypertrophy compared with ACE inhibitors or AT1R blockers in patients with HF with a reduced LV ejection fraction [35].

While the classical ACE/Ang II/AT1R pathway is considered to be deleterious when chronically activated by stimulating vasoconstriction, proliferation, and inflammation, the alternative ACE2/Ang 1-7/Ang 1-5/MasR seems to be a counterbalancing pathway by reducing oxidative stress and inflammation, inducing vasodilatation, and inhibiting myocyte growth and fibrotic proliferation [36,37,38,39]. In our experiment, ARNI, via AT1R blockade by valsartan, enhanced the levels of Ang II and Ang I and increased Ang 1-7 and Ang 1-5, along with Ang III and Ang IV. The marker of ACE activity (ACE-S), calculated as the Ang II/Ang I ratio, provides information about the expected ACE activity. ACE-S was lower in SHR and remained unaffected by ARNI. On the other hand, Ang 1-7 was remarkably increased by ARNI in SHRs and also stabilised, as shown by a trend to a reduced Ang 1-5/Ang 1-7 ratio. Ang 1-7 is considered to be a decisive player in damaged heart protection [40]. The increase in Ang 1-7 levels has to be considered with regard to the simultaneous AT1R blockade by valsartan, rendering Ang II ineffective and Ang 1-7 as the dominant effector in the RAAS. The results on serum aldosterone levels are puzzling. Although the AT1R blocker moiety of ARNI effectively blocked AT1R signalling in the adrenal glands, as indicated by a decreased aldosterone/Ang II ratio (AA2 ratio), the actual aldosterone level was increased by ARNI in SHRs, suggesting an important role of stimuli (such as potassium levels, NO availability, ACTH release or sympathetic activity) [41,42] for aldosterone production different from Ang II. Thus, in SHRs, the presumably protective effect of increased Ang 1-7 may be partly counterbalanced by elevated aldosterone levels. The increased serum levels of aldosterone with ARNI treatment might have determined the only mild-to-moderate antifibrotic effect of ARNI in this study. One could hypothesise that the potential combination of ARNI with a mineralocorticoid receptor antagonist would be a beneficial strategy in the treatment of hypertensive hearts. Additionally, the inhibition of neprilysin by sacubitril, with the subsequent enhancement of ANP and BNP exerting vasodilative and antiproliferative effects [43], might have also contributed to the protection by ARNI in SHRs.

Elevated HR is a risk factor in healthy individuals and various cardiovascular pathologies [44]. Ivabradine selectively inhibits the I_f_ current of the pacemaker cells in the sinoatrial node, thus reducing HR without negative inotropy [45]. Although HR reduction coming with an improvement in myocardial energy balance seems to be the principal factor underlying the protection offered by ivabradine in HF patients, a number of pleiotropic effects, including antioxidative, anti-inflammatory, and neurohumoral action modulation, may contribute to heart protection [45]. Indeed, in aortic constriction-induced LV hypertrophy in mice, ivabradine attenuated LV hypertrophy, fibrosis, and dysfunction [46]. In isoproterenol-induced heart damage, ivabradine reduced LV fibrotic remodelling and improved survival [47]. In our previous experiment with L-NAME-induced hypertension, ivabradine improved the systolic and diastolic function of the remodelled LV [48]. In line with the above data, in this experiment, ivabradine reduced fibrosis of the LV and improved systolic and diastolic function in SHRs. However, ivabradine did not affect the serum concentrations of Ang II and Ang 1-7 or other downstream products of the classical and alternative pathways of the renin–angiotensin system. Interestingly, even the serum levels of aldosterone, which were reduced by ivabradine in L-NAME-induced hypertension [48] and supposedly contributed to the hypertensive heart protection by ivabradine, were unchanged in SHRs. There are two potential factors underlying the apparent protection by ivabradine in SHRs to consider. First, HR reduction associated with an improvement of myocardial energy balance in a haemodynamically overloaded hypertensive heart could result in improved LV contractility and relaxation. Second, ivabradine may provide a benefit via its presumable pleiotropic sympatholytic effect. Indeed, the pre-treatment of rats exposed to handling stress with ivabradine was associated with a reduced release of adrenaline and noradrenaline into the blood stream [49], and changes to heart rate variability (HRV) in a rat model of doxorubicin-induced HF indicated an improved autonomic imbalance by ivabradine [50]. The HRV analysis in a SHIFT Holter sub-study showed an ivabradine-mediated shift toward a more prominent parasympathetic tone [51].

Limitations: For the pathophysiological implications, the local tissue concentration of RAAS peptides remains important. It seems, however, that the local RAAS remains largely dependent on the circulating RAAS peptides, as previously discussed for the brain RAAS [52]. In fact, the concentration of renin and angiotensinogen in the heart tissue is very low compared to plasma [53]. Additionally, we have recently shown [48] that apart from Ang 1-10 and Ang 1-8, the tissue concentrations of other RAAS components remain low and the LV concentration of Ang II correlates with the circulating Ang II levels.

## 5. Conclusions

We conclude that the SHR is a normal-to-low RAAS model of experimental hypertension. Recent drugs from the portfolio of HF management—ARNI and ivabradine—exerted the attenuation of LV remodelling and dysfunction in the SHR. Considering the changes to the RAAS, the cardiovascular protection by ARNI may be related to the Ang II blockade and the protective nature of Ang 1-7, while the cardiovascular protection by ivabradine was not associated with the modification of RAAS in the SHR. 

## Data Availability

Data supporting the reported results are available from the corresponding author per request.
